# Evolution of dispersion-engineered metasurfaces: Debye relaxation and folded path concept

**DOI:** 10.1038/s41377-025-01890-2

**Published:** 2025-06-24

**Authors:** Hammad Ahmed, Buxiong Qi, Xianzhong Chen

**Affiliations:** 1 Institute of Photonics and Quantum Sciences, School of Engineering and Physical Sciences, Heriot-Watt University, Edinburgh, EH14 4AS UK; 2 School of Information Science and Engineering, Lanzhou University, 730000 Lanzhou, China

**Keywords:** Nanophotonics and plasmonics, Sub-wavelength optics

## Abstract

Dispersion engineering is advancing with recent breakthroughs in metasurfaces using the 2nd-order Debye relaxation and the folded-path concept, greatly improving relevant applications such as imaging, beam shaping, and cloaking.

## Graphical abstract

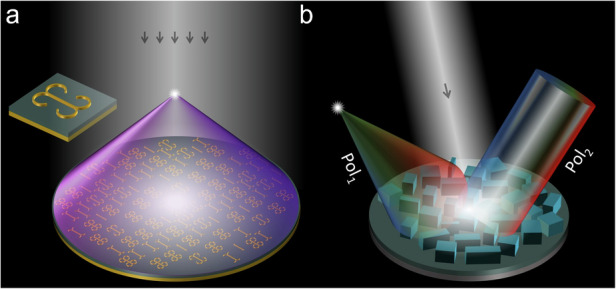

Dispersion arises from the change in a material’s refractive index based on the frequency of incident electromagnetic waves. In contrast to conventional refractive devices, the dispersion, effective permittivity, permeability, and refractive index of metasurfaces consisting of subwavelength meta-atoms are primarily influenced by their geometric configuration and arrangement rather than their material properties. Recently, the dispersion properties of metasurfaces have been used as useful tools to demonstrate various applications such as spectrometers^1,2^, multicolored optical knots^3^, 3D curved trajectories^4^, and multispectral polarization states^5^. However, accurate dispersion control is crucial in many applications. For example, it can mitigate chromatic aberrations in imaging systems^6^, improve virtual reality displays^7^, and reduce pulse spreading in optical fibers^8^.

Numerous attempts have been made to achieve precise dispersion control, often involving multi-layered structures or complex geometries^9–12^. This article focuses on two recent papers with new physics published in *Light: Science & Applications*^13^^,^^14^. The first paper, in the microwave regime, employs the 2nd-order Debye relaxation to achieve smooth phase transitions, enhancing applications such as wideband beam deflectors and achromatic focusing. The second, in the terahertz range, utilizes folded-path metasurfaces to realize independent dispersion control for two circular polarization states, tackling a fundamental challenge in spin photonics without the need for complex structural design.

In the paper titled “2nd-Order Debye Relaxation in Electromagnetic Metasurfaces for Wideband Dispersion Engineering”, a team led by Professors Jiafu Wang, Jie Yang and Tiejun Cui proposed using the 2nd-order Debye relaxation in electromagnetic metasurfaces to enable the wideband dispersion engineering as shown in Fig. 1a. Traditionally, Drude and Lorentz models are used in metamaterials to describe electromagnetic properties, leaving a gap between dielectric physics and metamaterials since the Debye model, crucial for dielectric relaxation, was largely neglected. This research addresses this gap by demonstrating that metasurfaces can exhibit the higher-order Debye relaxation through tailored resonances. The study first analyzes fundamental resonance modes of meta-atoms, revealing that magnetic and electric resonances—typically Lorentzian—can be tuned to form a 2nd-order Debye relaxation. This phenomenon enables smooth phase variations between resonance frequencies, making it ideal for wideband dispersion engineering. A quad-elliptical-arc meta-atom structure is proposed to achieve the 2nd-order Debye relaxation (Fig. 1a inset). The study demonstrates the wideband capabilities of metasurfaces using two prototypes: one for chromatic focusing and the other for achromatic focusing within the X-band frequency range (8.0–12.0 GHz). Simulation and experimental results verify that these metasurfaces achieve efficient dispersion management, enabling wideband beam deflection and planar focusing. This approach can potentially be extended to other frequency ranges like terahertz and optical frequencies, offering new opportunities for applications in radar systems, holographic imaging, and next-generation communication technologies.

**Fig. 1:**
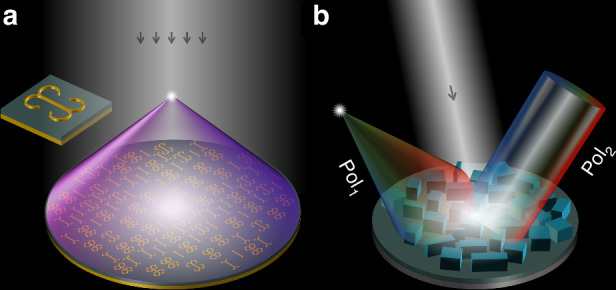
Schematic of the dispersion-engineered metasurfaces. **a** Metasurface based on the 2nd-order Debye relaxation. **b** Folded-path metasurface

In the other paper titled “Dispersion-engineered spin photonics based on folded-path metasurfaces”, the team led by Professors Mingbo Pu and Xiangang Luo presented a novel approach to dispersion-engineered spin photonics using folded-path metasurfaces (Fig. 1b). These metasurfaces enable the independent control of dispersion and phase for two opposite spin states (Pol_1_ and Pol_2_), overcoming previous limitations in achieving broadband decoupling and integration. The innovation lies in folding the optical path via the local interference at the subwavelength scale, creating a virtual reflective surface that adjusts the effective path length. Unlike traditional methods that rely on changing the structural geometry or height, this approach achieves dispersion control without altering the metasurface height. The folded-path metasurface concept is demonstrated with the polarization-decoupled interference, allowing versatile wavefront shaping and independent dispersion control for any pair of orthogonal polarization states. This method supports advanced applications like achromatic focusing, achromatic photonic spin Hall effect PSHE, and spatiotemporal vector optical field generation. Experimental demonstrations validate the concept, including an achromatic metalens operating in the 195–225 THz range. The metalens achieves achromatic focusing without varying geometrical sizes, utilizing fixed-dimension supercells with rotational adjustments. Additionally, the metasurface demonstrates broadband achromatic PSHE and spatiotemporal vector optical field manipulation, showcasing robust and consistent performance over a wide frequency range. This folded-path metasurface platform revolutionizes spin photonics by providing unprecedented control of spin-decoupled dispersion and phase using a single metasurface layer. The concept can be extended to various wavelength ranges, paving the way for next-generation spin-photonic devices with applications in dynamic light-matter interaction, information encoding, and compact optical systems.

Both studies have successfully achieved dispersion engineering with a single metasurface. The approaches developed by both research groups demonstrate the remarkable flexibility in designing optical elements with nearly arbitrary spatial phase profiles and dispersion properties, without the need for additional optical components or materials. This is a significant advantage as it enables compact design while keeping fabrication costs comparable to those of conventional dispersive metasurfaces. The two dispersion-engineered metasurfaces will impact imaging, beam shaping, and cloaking while driving innovations in electromagnetic devices. For example, the folded metasurfaces can be used to realize invisibility carpet cloaking, where precise control over light propagation can render objects invisible across multiple wavelengths. However, it is important to note that both studies are based on reflection-type metasurfaces, which are incompatible with most transmission-type systems.
